# Supplementary material for: Comprehensive Genome-Wide Analysis of the Receptor-like Protein Gene Family and Functional Analysis of PeRLP8 Associated with Crown Rot Resistance in Passiflora edulis
Source: Plants (Basel). 2024 Nov 21;13(23):3264. doi: 10.3390/plants13233264 (PMC11644041; doi:10.3390/plants13233264)
Supplement: Supplementary file 1 [file plants-13-03264-s001.zip › plants-3275818-supplementary.pdf]

Table S1 Primers for real-time quantitative PCR, gene full length cloning, and vector construction.

Genes	Primer sequence	Used for
q-*PeRLP8*-F	AGCACGCTGAAACTGGCT	qRT-PCR
q-*PeRLP8*-R	CTTGGAACCTGGCCAGCA	
q-*PeRBOHD*-F	CAGGCAGGTTTCGCAGGA	
q-*PeRBOHD*-R	GAGGCTCCGGTTTTGGCT	
q-*PeMYC*-F	CCACGGGACTCCACCAAC	
q-*PeMYC*-R	CAAGACGAACCGACCGCT	
q-*PeJAR1*-F	GCTCTACCTCGCCTCCCT	
q-*PeJAR1*-R	CGAGCCGTACAACCTCCTC	
PeRLP8-F	ATGAAAATTCCTCTGTTATTTACATGGGTTGTC	clone
PeRLP8-R	TTATGAAGAAGAGGAAGAGAAGGAAGAAGAG	
PeRLP8-GFP-F	ACGGGGGACTCTTGACCATGG	
	ATGAAAATTCCTCTGTTATTTACATGG	
PeRLP8-GFP-R	AAGTTCTTCTCCTTTACTAGT	
	TGAAGAAGAGGAAGAGAAGGAAGAA	

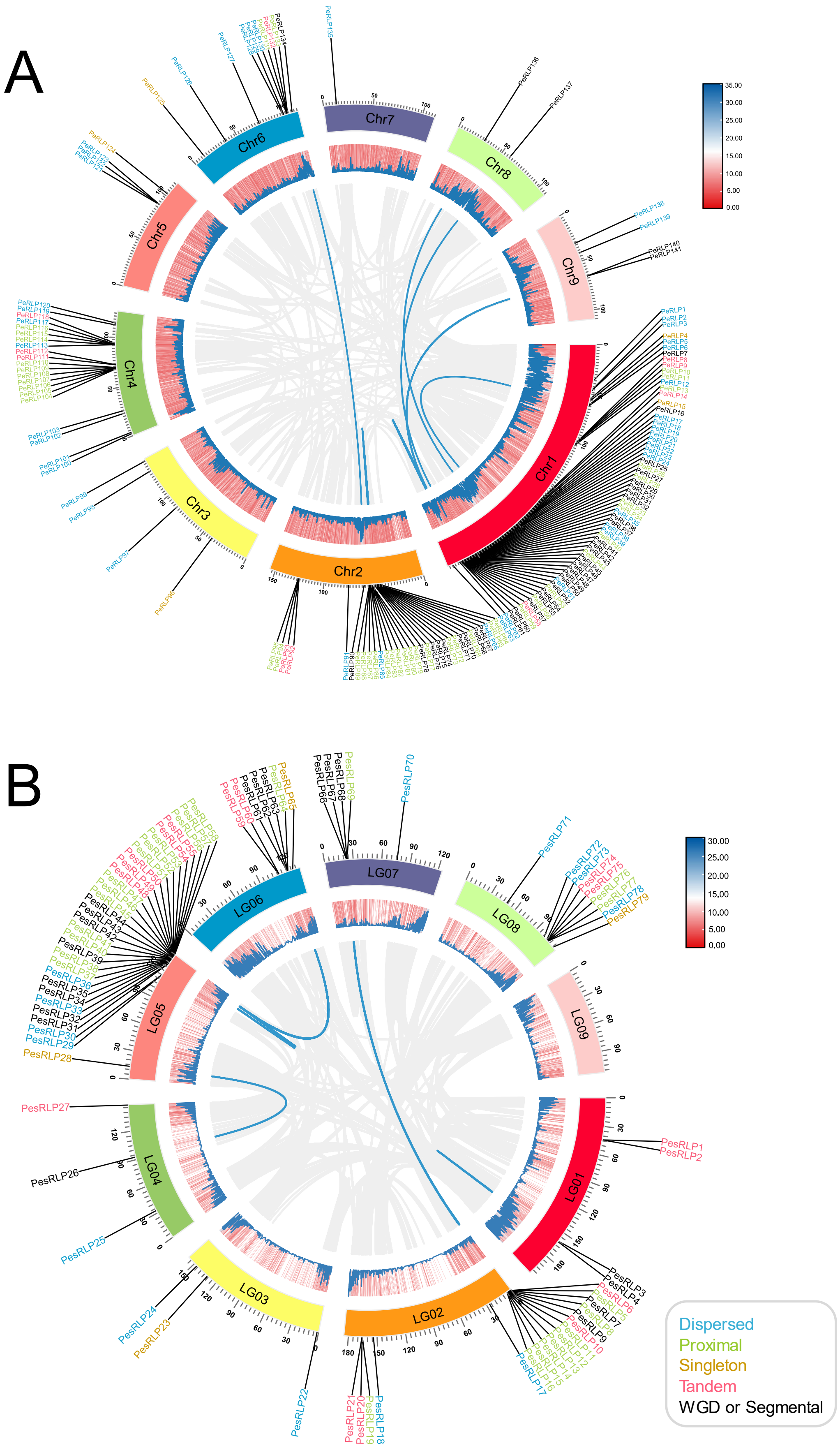

A
Chr1
Chr2
Chr3
Chr4
Chr5
Chr6
Chr7
Chr8
Chr9
B
LG01
LG02
LG03
LG04
LG05
LG06
LG07
LG08
LG09
PesRLP1
PesRLP2
PesRLP22
PesRLP23
PesRLP24
PesRLP25
PesRLP26
PesRLP27
PesRLP28
PesRLP70
PesRLP71
Dispersed
Proximal
Singleton
Tandem
WGD or Segmental

Figure S1 Identification and evolutionary analysis of *RLP* family genes in passion fruit. (**A**) Chromosomal localization and collinearity analysis of 141 *PeRLP* genes in the ZX genome. (**B**) Chromosomal localization and collinearity analysis of 79 *PesRLP* genes in the TN genome.

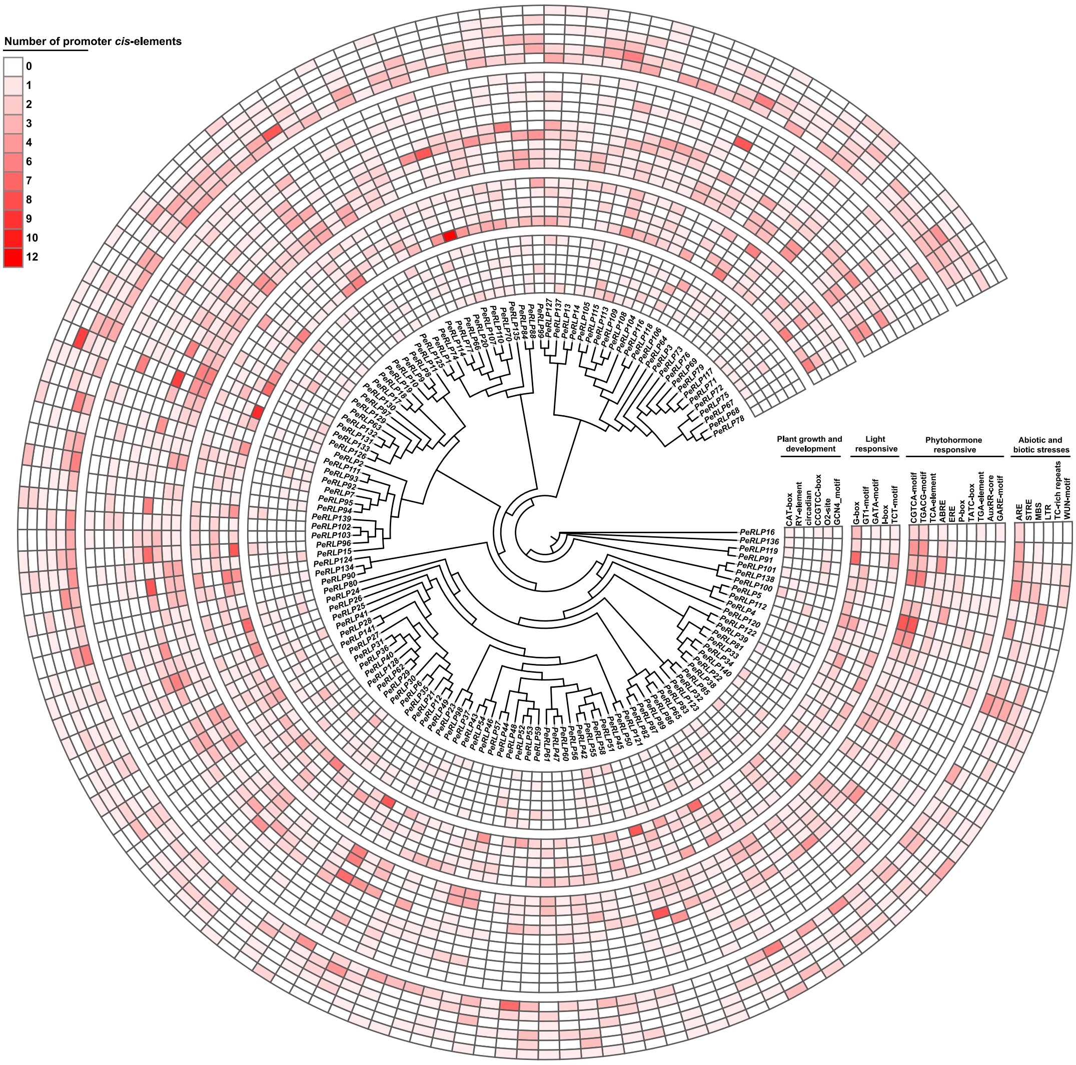

Figure S2 The cis-regulatory elements in promoters of *PeRLP* family genes. The number of different cis-regulatory elements in the promoter was represented by square bars of different colors.

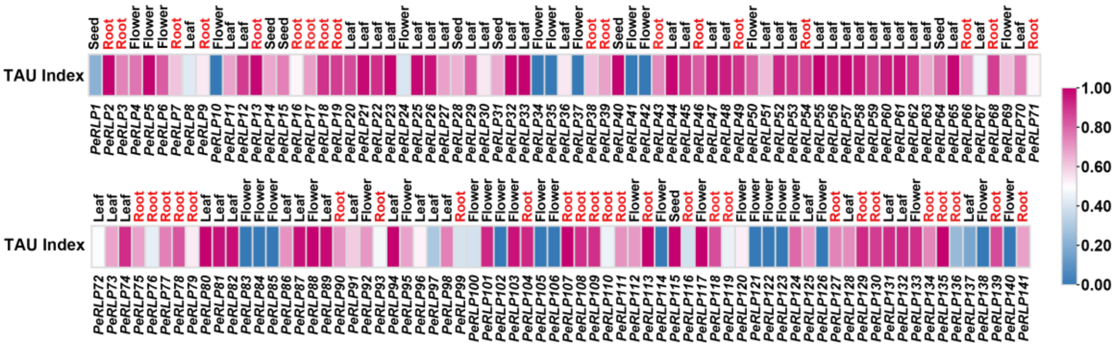

Figure S3 Tissue specific calculations of 141 *PeRLP* genes in root, leaf, seed, and flower. Differences in gene expression changes were shown in color as the scale, mediumvio-letred for high expression and steelblue for low expression.

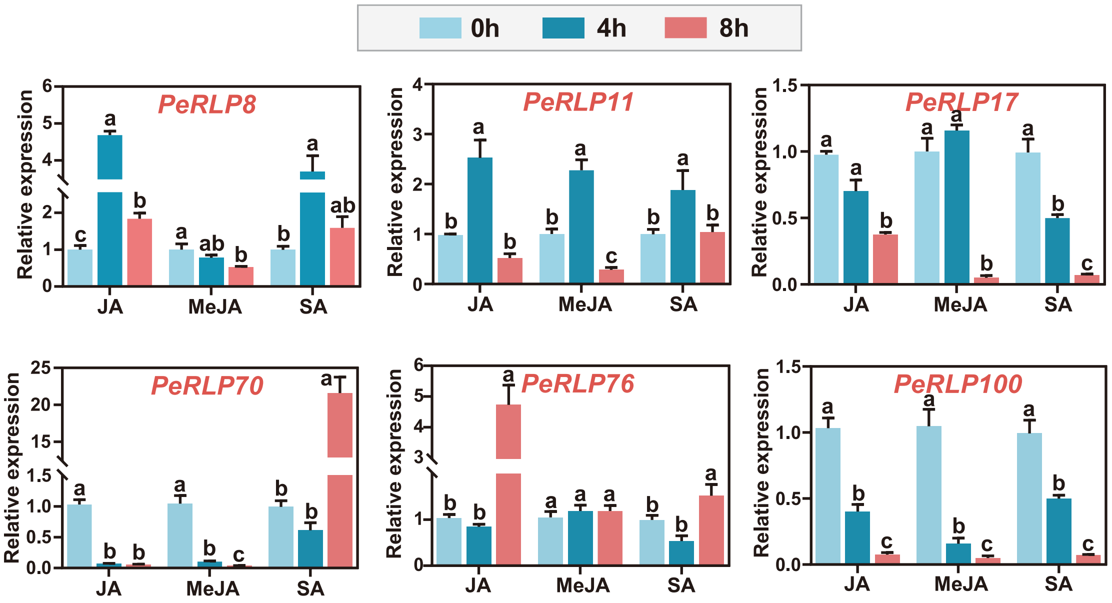

Figure S4 The expression levels of 6 *PeRLP* genes at 0h, 4h and 8h after JA, MeJA and SA treatment, respectively.

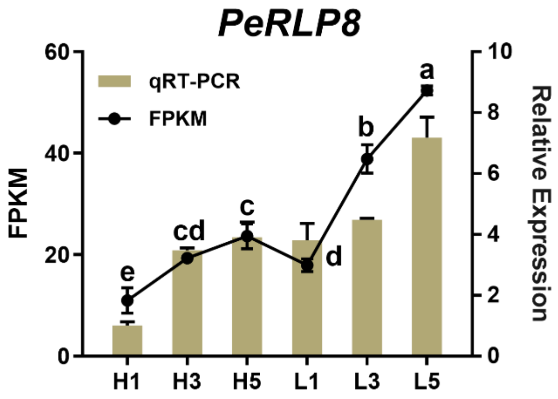

Figure S5 Quantitative detection of *PeRLP8* gene in resistant variety LG and sensitive variety HG infected by *R. solani*. The data presented are the mean values ± standard deviation (SD) from three independent replicates. Different letters denote significant differences (Student's t-test, $P < 0.05$).
